# Delayed unilateral eyelid oedema following non-periocular hyaluronic acid injection: A case report and literature review

**DOI:** 10.1016/j.jpra.2024.09.017

**Published:** 2024-09-27

**Authors:** Cecilia Soldini, Luigi Schiraldi, Pietro Giovanni Di Summa

**Affiliations:** a Department of Maxillofacial Surgery, Lausanne University Hospital (CHUV), Switzerland; b Department of Plastic and Hand Surgery, Lausanne University Hospital (CHUV), Switzerland; c Department of Plastic and Hand Surgery, Lausanne University Hospital (CHUV), Université de Lausanne (UNIL), Switzerland

**Keywords:** Hyaluronic acid, Glabellar filler, Peri-ocular oedema, Hyaluronidase, Filler complications

Hyaluronic acid (HA) is a non-sulphated glycosaminoglycan, and the main element of the skin's extracellular matrix. It is the most common soft tissue filler worldwide, thanks to its biocompatibility, accessibility, and rheological properties allowing very precise corrections and making it suitable for small areas like the face. Because of its popularity, physicians must be trained in recognising and treating HA-related complications, rare but increasing.^1^, ^2^, ^3^, ^4^

To the best of our knowledge, all described cases of peri‑ocular or malar HA-related oedema stem from tear-trough and eyelid deformities corrections. We report a case of delayed unilateral eyelid oedema following glabellar lines correction, a first (Figure 1).

**Figure 1:**
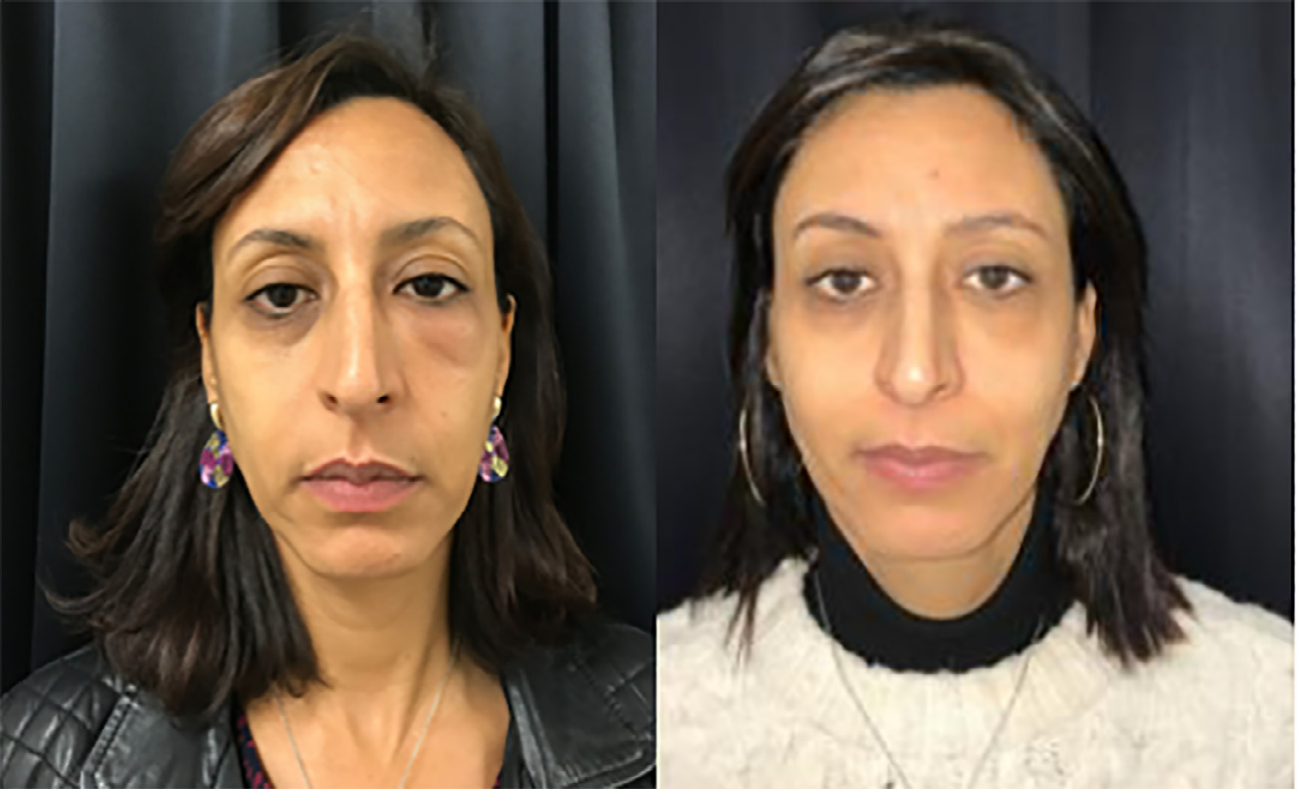
Before any treatment, and after third hyaluronidase injection.

A healthy 47-year-old woman referred to the emergencies for a left lower lid and malar oedema of overnight appearance. Complete ocular examination and blood work were strictly normal. She did not take any medications or drugs and denied any ongoing or recent cosmetic procedure.

An MRI showed infiltration of the left infero-lateral extraconal fat tissue of the eye, evocative of a reaction to HA.

Upon a more detailed anamnesis, the patient admitted to a past HA injection to correct glabellar lines 7 months prior.

Suspecting a migration of the filler, we decided to do a test treatment of the left lower eyelid with a small dose of hyaluronidase (300 UI) into the oedematous tissues, after a negative immunogenic test. Follow-up at 4 weeks showed clear improvement, with some residual swelling. The treatment was repeated with an increased dose (900 UI) twice, at 4-week intervals.

Despite the oedema being dramatically reduced, the patient still reported propensity of the left lower eyelid for morning swelling.

A longer follow up is needed to determine whether the condition is permanent, and observation, further treatment with hyaluronidase, or surgery are valid options to consider.

Knowledge of the rheology of the filler helps in understanding and preventing the possible complications. HA is a viscoelastic gel, sold in a combination of cross-linked (insoluble and more resistant to enzymatic degradation) and free form (soluble and less long-lasting). Elasticity, the force (G’) that allows the gel to return to its original shape after the injection, cohesivity and cross-linking are the main properties conferring a filler its firmness and lifting effect.^4^ When low, the filler tends to be degraded rapidly, and suffers a gravitational pull with a risk of migration, that can be favoured by muscle activity, high volume injection, and vigorous massage.^2^^,^^3^

Minor early complications (bleeding, haematoma, oedema, erythema, and allergic reactions) usually appear at the injection site hours to days after the injection, and are self-limiting. They are related to poor injection technique and filler selection: excess volume or repeated sessions, retro-septal or superficial placement, and use of less cohesive HA are risk factors.^1^^,^^5^ Major acute complications such as microvascular compression or thrombosis are fortunately rare, but potentially leading to necrosis, or blindness when occurring in the high-risk periocular region. They can be prevented by good knowledge of the surrounding anatomy.^1^^,^^2^^,^^4^

Late complications (>1 month after the injection) include the Tyndall effect, immune-inflammatory reactions such as granulomas and inflammatory nodules (with a compatible histology when biopsied) and HA migration.^1^^,^^2^^,^^5^ Amongst these, late-onset oedema, almost exclusive to the periorbital region, is poorly understood, and can appear years after the first injection. Because of this latency, the causal link with the injection is not always clear, and the patient undergoes multiple inconclusive diagnostic investigations, consulting with multiple specialists.^1^ In front of chronic periorbital oedema of undetermined origin, one should always investigate past HA injections in peri‑ocular or distant areas, even years prior.

Such persistent effect of the filler is multifactorial. HA metabolism shows progressive molecular degradation, the rapidity inversely proportional to the cross-linking ratio, each new degraded molecule retaining further water in an isovolumetric degradation fashion, contributing to the late appearance and persistence of the oedema, longer than the 6–12 months described. Use of high G’ fillers with increased hydrophilic properties and superficial injections also favour the oedema, as well as immune activation from antigenic stimulation.^1^^,^^2^^,^^3^^,^^5^

The simultaneous treatment with botulinum toxin can worsen the swelling, lowering the lymphatic pump-like activity of the orbicularis oculi. Injection directly into the muscle has the same effect, with histologic evidence of degeneration of the fibers. Past blepharoplasty also interferes with the normal drainage of the eye.^1^^,^^2^^,^^5^^,^^6^

To manage late-onset peri‑ocular oedema, no clear protocol is described, the therapeutic options being extrapolated from the few similar cases. Observation is unsatisfactory, the oedema worsening over time. Surgery is a valid option, but after correction of the oedema to avoid excessive resection of tissues and overcorrection.^1^ Prompt hyaluronidase injection is the most satisfactory alternative, in a stepwise fashion with progressive higher doses to avoid over-depletion. Swelling can completely resolve, but persistence of the effect over the long-term needs to be determined. No side effects of hyaluronidase are reported to date.^3^^,^^5^

Because of a possible immune component, non-steroidal anti-inflammatory drugs (NSAID) and oral or topical steroids could be beneficial, as prevention or treatment, but more robust supportive results are needed, given the potential side effects.

In front of residual oedema after hyaluronidase treatment, as in our patient, observation, further hyaluronidase injections, oral/topical NSAID or steroids and surgery are all valid options, with no established sequence or evidence of superiority of one, nor long-term follow-up data available. Well-structured studies with a longer follow-up are needed to determine a clear therapeutic protocol for delayed complications of HA injection.

## Declaration of competing interest

The authors declare no conflicts of interests.
