# Supplementary figures and images for: Delayed unilateral eyelid oedema following non-periocular hyaluronic acid injection: A case report and literature review
Source: JPRAS Open. 2024 Sep 27;42:334–7. doi: 10.1016/j.jpra.2024.09.017 (PMC11566713; doi:10.1016/j.jpra.2024.09.017)

**Supplement:** head MRI showing left peri-orbital HA reaction.

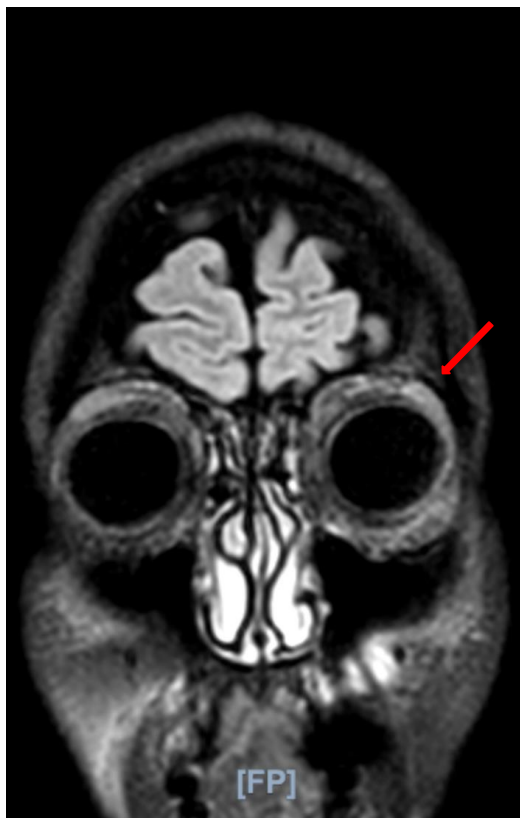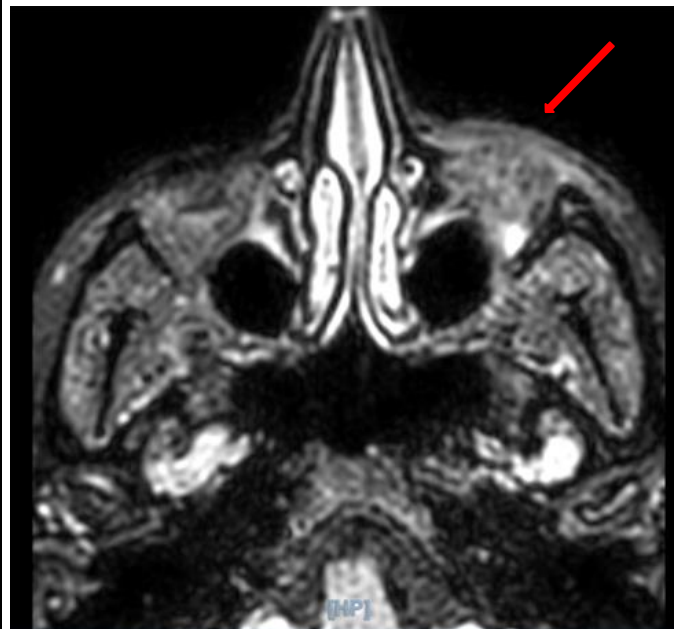

Supplement: Supplementary Data S1 [file mmc1.pdf]
